# Nonlinear association between glycated hemoglobin levels and mortality in elderly patients with non-diabetic chronic kidney disease: a national health and nutrition examination survey analysis

**DOI:** 10.3389/fendo.2025.1416506

**Published:** 2025-02-11

**Authors:** Lihua Huang, Liuliu He, Qingfeng Zeng, Jinjing Huang, Xiaoyan Luo, Qiuming Zhong

**Affiliations:** ^1^ Department of Clinical Laboratory, The Second Affiliated Hospital of Gannan Medical University, Ganzhou, China; ^2^ Department of Neurology, The Second Affiliated Hospital of Gannan Medical University, Ganzhou, China; ^3^ Department of Gastroenterology, The Second Affiliated Hospital of Gannan Medical University, Ganzhou, China; ^4^ Department of Interventional Radiology, The Second Affiliated Hospital of Gannan Medical University, Ganzhou, China; ^5^ Department of Hospital Pharmacy, The Second Affiliated Hospital of Gannan Medical University, Ganzhou, China

**Keywords:** chronic kidney disease, non-diabetic, mortality, HbA1c, NHANES

## Abstract

**Background:**

The relationship between glycated hemoglobin (HbA1c) levels and mortality outcomes in elderly patients with non-diabetic chronic kidney disease (CKD) has not been well characterized. This study aimed to investigate the correlation between HbA1c levels and all-cause and cardiovascular disease (CVD) mortality in elderly individuals with non-diabetic CKD.

**Methods:**

Data from the NHANES (1999-2018) were analyzed to measure HbA1c levels in whole blood using high-performance liquid chromatography (HPLC). Information on deaths and subsequent details was collected through the National Mortality Index until December 31, 2019. Hazard ratios (HR) and 95% confidence intervals (CIs) for all-cause and CVD mortality were calculated using weighted Cox proportional hazards and restricted cubic spline models.

**Results:**

Among the 1,931 participants (mean [SE] age, 73.2 [0.2] years; 61.9% female), over a median follow-up period of 7.6 years, a total of 1,003 deaths were observed, including 412 from CVD. HbA1c was divided into four quartiles: Quartile 1 (3.7–5.3%), Quartile 2 (5.4–5.6%), Quartile 3 (5.7–5.8%) as the reference group, and Quartile 4 (5.9–6.4%). Higher risks of all-cause mortality were noted in the lowest and highest HbA1c quartiles, with adjusted HR (95% CI) of 1.48 (1.18–1.87) and 1.31 (1.01–1.70) respectively. For CVD mortality, the lowest quartile showed a significantly increased risk (HR 1.94, 95% CI: 1.29–2.90), but the highest quartile did not significantly differ from the reference, with HR 1.14 (0.73–1.77). The RCS analysis indicated a U-shaped nonlinear relationship between HbA1c levels and all-cause mortality (P = 0.026 for nonlinearity) and a J-shaped nonlinear relationship with CVD mortality (P = 0.035 for nonlinearity).

**Conclusion:**

This cohort study suggests that both low and high HbA1c levels are associated with an increased risk of all-cause mortality in elderly patients with non-diabetic CKD.

## 1 Introduction

The Global Burden of Disease (GBD) study revealed that chronic kidney disease (CKD) was the 16th most common cause of death in 2016 and is projected to rise to the 5th position by 2040 (1). Older adults represent a significant and growing proportion of the individuals receiving treatment for kidney disease. Advanced age is a key risk factor for the development of cardiovascular disease (CVD) and the primary cause of death in patients with CKD (2), with mortality increasing as CKD progresses (3). In patients with diabetic nephropathy, effective glycemic control has demonstrated a notable reduction in cardiovascular events, slowing CKD progression, and improving prognosis (4). However, the relationship between glycemic levels and long-term outcomes in patients with non-diabetic nephropathy remains unclear.

Glycated hemoglobin (HbA1c), a crucial marker for glycemic management, is used to assess average blood glucose levels over approximately 3 months (5). The National Kidney Foundation Kidney Disease Outcomes Quality Initiative (NKF–KDOQI) and Kidney Disease Clinical Practice Guidelines recommend HbA1c for assessing glycemic control (6). For non-diabetic patients, elevated HbA1c levels are associated with an increased risk of CKD (7, 8). Studies have confirmed an association between high HbA1c levels and coronary heart disease, stroke, and overall mortality in the non-diabetic population, often depicted as a J-curve (9) or U-shaped relationship (10). However, managing glycemia in non-diabetic nephropathy becomes complex due to CKD’s effects, including reduced β–cell response, decreased renal insulin clearance, and increased hepatic gluconeogenesis (11). To date, there have been few studies on the association between HbA1c and mortality outcomes in non-diabetic patients with CKD, and the conclusions of the limited studies are inconsistent (9, 12, 13). In addition, existing studies have ignored the factors commonly reported to affect HbA1C, such as CKD stage and anemia, and the main populations of the studies have been young and middle-aged. Considering that elderly patients with CKD have a higher risk of mortality, we aimed to investigate the association of HbA1c levels with all-cause and cardiovascular deaths in elderly patients with non-diabetic CKD using data from the National Health and Nutrition Examination Survey (NHANES).

## 2 Materials and methods

### 2.1 Study design and population

**Figure 1:**
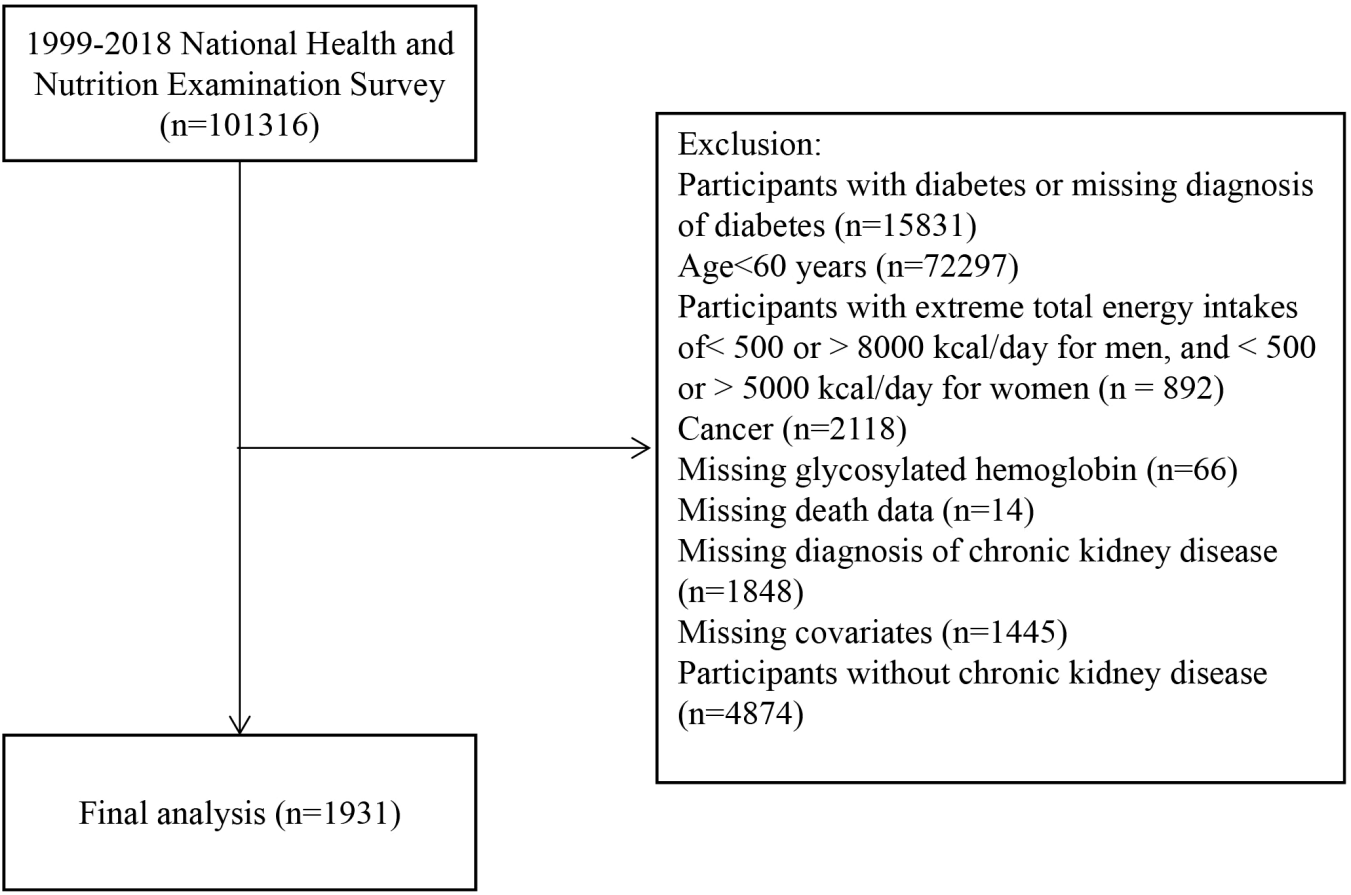
Study flowchart.

The data for this investigation were sourced from the NHANES, a forward–looking cohort investigation aimed at evaluating the health and nutritional status of individuals in the United States. The methodology for the analysis has been detailed in a previously published study (14). The NHANES, which was overseen by the National Center for Health Statistics, a branch of the Centers for Disease Control and Prevention (CDC), received ethical clearance from the Institutional Review Board of the National Center for Health Statistics. All participants provided written informed consent to participate in this study. This study adhered to the Strengthening the Reporting of Observational Studies in Epidemiology (STROBE) Cohort Study reporting guidelines. Data from ten NHANES cycles (1999–2018) were used in this study. Patients were excluded based on specific criteria: those with a diabetes diagnosis (defined as having a doctor’s diagnosis, insulin or glucose-lowering medication use, a HbA1c level of 6.5% or higher, or a fasting blood glucose level of 126 mg/dL or higher) or missing diagnostic information on diabetes at baseline (n = 15,831), individuals aged <60 years (n = 72,297), participants with extreme energy intake (n = 892; these values may result from altered metabolism or comorbidities in elderly CKD patients and could significantly impact analyses, thus excluding <500 or >5000 kcal/day for women, <800 or >8000 kcal/day for men), individuals with cancer at baseline (n = 2,118), those with missing HbA1c data (n = 66), missing mortality status (n = 14), missing covariates (n = 1,455), missing CKD diagnostic information (n = 1,848), and those not diagnosed with CKD (n = 4,874). After applying these exclusion criteria, the analysis included 1,931 individuals aged ≥60 years with non-diabetic CKD (**Figure 1**).

### 2.2 Measurement of glycated hemoglobin

HbA1c levels were quantified in whole blood samples through high–performance liquid chromatography (HPLC), utilizing instruments accredited by the National Glycated Hemoglobin Standardization Program and aligned with the reference methodology of the Diabetes Control and Complications Trial. The participants were stratified into four groups based on HbA1c levels. While quartile categorization typically results in equal group sizes, the natural clustering of HbA1c values in our population led to an uneven distribution across the groups. According to standard statistical procedures, group assignment is defined as “lower bound < X ≤ upper bound,” with values equal to the cutpoint assigned to the higher group. The final categorization was as follows: Quartile 1 (3.7-5.3%, n=484), Quartile 2 (5.4-5.6%, n=632), Quartile 3 (5.7-5.8%, n=385), and Quartile 4 (5.9-6.4%, n=430). The larger sample size in Quartile 2 reflects the natural clustering of HbA1c values around 5.4-5.6% in our study population, a phenomenon commonly observed when categorizing clinical laboratory values, which often cluster around specific ranges.

### 2.3 Definition of CKD

In this study, CKD was identified based on the urinary albumin-to-creatinine ratio (ACR) and estimated glomerular filtration rate (eGFRs). CKD was defined by an eGFR <60 mL/min/1.73 m^2^ or a ACR ≥30 mg/g according to KDIGO guidelines (15). The ACR categories were divided into <30 mg/g (A1), 30–300 mg/g (A2), and >300 mg/g (A3) based on urinary tests. The eGFR was calculated using the CKD-EPI 2009 (Chronic Kidney Disease Epidemiology Collaboration) creatinine equation. The eGFR categories were G1 (≥90 mL/min/1.73 m^2^), G2 (60–89 mL/min/1.73 m^2^), G3a (45–49 mL/min/1.73 m^2^), G3b (30–44 mL/min/1.73 m^2^), G4 (15–29 mL/min/1.73 m^2^), and G5 (<15 mL/min/1.73 m^2^).

### 2.4 Covariates

Race/ethnicity were evaluated as fundamental demographic factors and classified according to self-report during the interview. Hypertension was defined as systolic and diastolic blood pressure measurements of at least 140 mm Hg and 90 mm Hg, respectively, self-reported high blood pressure, diagnosed by a doctor, or requiring medication to control elevated blood pressure. CVD was defined as self-reported diagnoses of congestive heart failure, angina, myocardial infarction, or coronary heart disease. Cancer data were self-reported after diagnosis by a physician. Hyperlipidemia was characterized by either a physician’s diagnosis or current use of cholesterol-lowering medication, or having any of these lipid parameters: elevated triglycerides (≥150 mg/dl; 1.7 mmol/L), increased total cholesterol (≥200 mg/dl; 5.18 mmol/L), high LDL cholesterol (≥130 mg/dl; 3.37 mmol/L), or reduced HDL cholesterol (men: <40 mg/dl, 1.04 mmol/L; women: <50 mg/dl, 1.30 mmol/L) (16). Healthy Eating Index (HEI) scores were calculated according to HEI-2015 guidelines. Alcohol consumption was categorized as never, former, moderate, mild, or heavy based on detailed explanations provided earlier (17). Smoking status was categorized into current smokers, former smokers, or never smokers based on participants’ responses to a series of questions. Physical activity was defined as engaging in moderate to vigorous sports, fitness programs, or recreational activities for more than 10 minutes per week; otherwise, participants were classified as inactive. Anemia was defined as hemoglobin levels below 120 g/L in females and below 130 g/L in males according to WHO criteria (18). The neutrophil-to-lymphocyte ratio (NLR) was calculated by dividing the number of neutrophils by the number of lymphocytes.

### 2.5 Mortality outcomes of the study population

Mortality data were obtained from the National Death Index (NDI) database maintained by the Centers for Disease Control and Prevention (CDC). Each participant was followed from enrollment until their death or December 31, 2019, the final update of the NDI database. Only 14 participants were lost to follow-up due to missing mortality data, representing less than 1% of our study population. Instances of cardiovascular mortality were identified using the ICD-10 codes (I00-I09, I11, I13, and I20-I51) from the International Statistical Classification of Diseases, 10th Revision (ICD-10).

### 2.6 Statistical analysis

Following NHANES guidelines, this study adjusted for complex sampling designs and weights, reporting weighted averages for continuous variables and percentages for categorical ones. All continuous variables were analyzed without transformation prior to analysis. ANOVA or the Kruskal-Wallis test compared continuous variables, while the chi-square test assessed categorical data differences. For the non-diabetic CKD population, a restricted cubic spline (RCS) with four knots was used to explore the potential nonlinear correlation between HbA1c levels and mortality from all causes and CVD. The number of knots was selected based on model fit assessment using Akaike Information Criterion (AIC), with the restricted cubic spline (RCS) regression performed using 4 knots at the 5th, 35th, 65th, and 95th percentiles, demonstrating optimal performance. Survey-weighted Cox regression analysis was employed to evaluate the impact of HbA1c on overall CVD mortality. An exploratory analysis was conducted with Quartile 3 (5.7–5.8%) selected as the reference group due to its lowest associated risk of mortality. Three models were developed to adjust for potential confounders. Model I included age (continuous) and sex (male or female). Model II further adjusted for race/ethnicity (other Hispanic, Mexican American, non-Hispanic black, non-Hispanic white, or other, including multiracial), education level (<9 years, 9–13 years, or ≥13 years), poverty-to-income ratio (PIR) (continuous), BMI (continuous), smoking status (never, former, or current smoker), and comorbidities (hypertension, CVD, hyperlipidemia), as well as physical activity (inactive or active) and alcohol intake (never, former, moderate, mild, heavy). Model III additionally accounted for the HEI (continuous), hemoglobin (continuous), albumin (continuous), neutrophil-to-lymphocyte ratio (NLR) (continuous), and eGFR (continuous).

The analysis was stratified according to various factors such as sex (male or female), race/ethnicity (other Hispanic, Mexican American, non–Hispanic black, non–Hispanic white, or other, including multiracial), education level(<9 years, 9–13 years, or ≥13 years), PIR (<1, 1–3, ≥3), BMI (<30, ≥30kg/m^2^), smoking status (never, former, or smoker), hypertension (yes, no), hyperlipidemia (yes, no), CVD (yes, no), anemia (yes, no), eGFR categories (G1, G2, G3a+G3b, and G4+G5), and ACR categories (A1, A2, and A3). Interactions between HbA1c levels and these stratified factors were examined using P-values for the interaction terms.

Sensitivity analyses were conducted to address two potential sources of bias. First, to minimize reverse causality bias, we excluded participants who died within two years of follow-up. Second, because patients with end-stage renal disease (ESRD) may have HbA1c values that underestimate glycemic control due to uremia-related changes in hemoglobin conversion and carbamylation (19, 20), we excluded individuals with an estimated glomerular filtration rate (eGFR) <15 mL/min/1.73 m² and those who reported undergoing hemodialysis in the past year. Both sensitivity analyses used survey-weighted Cox regression, with full adjustment for all covariates in Model III.

Data management and statistical analyses were conducted using R version 4.3.1 (The R Foundation). Statistical analyses were performed using the following R packages: ‘survival’ for Cox models, ‘rms’ for restricted cubic splines, and ‘survey’ for complex survey analyses. A two-sided P value <0.05 was considered to be statistically significant.

## 3 Results

### 3.1 Study participants and baseline characteristics

A total of 1,931 participants were enrolled in the study, with a mean age of 73.2 years (standard error [SE] = 0.2) and comprised of 61.9% (n=1,034) females. The mean HbA1c level was 5.6% (SE 0.01). During a median follow-up of 7.6 years, a total of 1,003 deaths occurred, with overall survival rates of 83.9% at 5 years and 59.3% at 10 years. Baseline characteristics distributed across HbA1c Quartiles—Quartile 1 (3.7–5.3%), Quartile 2 (5.4–5.6%), Quartile 3 (5.7–5.8%), and Quartile 4 (5.9–6.4%)—are detailed in **Table 1**. Individuals with elevated HbA1c levels were more likely to be non–Hispanic Black, and were associated with lower BMI, former smoking, hypertension, higher eGFR categories, reduced physical activity, lower NLR, and decreased albumin and hemoglobin levels. The third Quartile exhibited the lowest mortality rate.

**Table 1:** Baseline characteristics of the study participants.

Variables		Hba1c (%)				
		3.7-5.3	5.4-5.6	5.7-5.8	5.9-6.4	
	Total (n = 1931)	Quartile1(n =484)	Quartile2 (n = 632)	Quartile3 (n = 385)	Quartile4 (n = 430)	P-value
	total	Q1	Q2	Q3	Q4	
Age, years, Mean ± SE	73.2 (0.2)	72.9 (0.5)	73.4 (0.3)	73.0 (0.6)	73.6 (0.4)	0.68
Sex, n (%)						0.57
Female	1034 (61.9)	251 (59.3)	341 (61.7)	206 (62.1)	236 (65.3)	
Male	897 (38.1)	233 (40.7)	291 (38.3)	179 (37.9)	194 (34.7)	
Race-ethnicity, n (%)						< 0.001
Mexican American	201 (2.6)	45 (1.9)	61 (2.2)	50 (3.8)	45 (2.9)	
Non-Hispanic Black	317 (7.4)	73 (6.0)	74 (4.9)	70 (8.5)	100 (12.0)	
Non-Hispanic White	1244 (84.4)	341 (88.4)	444 (87.9)	218 (79.2)	241 (78.1)	
Other Hispanic	95 (2.8)	14 (1.6)	33 (2.8)	28 (4.5)	20 (2.7)	
Other Race	74 (2.9)	11 (2.1)	20 (2.2)	19 (4.0)	24 (4.4)	
Poverty Income Ratio, Mean ± SE	2.8 (0.1)	3.0 (0.1)	2.8 (0.1)	2.6 (0.1)	2.7 (0.1)	0.12
BMI, kg/m^2^, Mean ± SE	28.2 (0.2)	26.9 (0.3)	28.3 (0.3)	28.7 (0.4)	29.3 (0.4)	< 0.001
Education Level, n (%)						0.64
Low (<9 years)	330 (9.6)	95 (10.4)	91 (8.4)	69 (11.0)	75 (9.5)	
Medium (9–13 years)	808 (42.4)	189 (39.7)	273 (42.5)	151 (41.6)	195 (46.2)	
High (≥13 years)	793 (48.0)	200 (49.9)	268 (49.1)	165 (47.5)	160 (44.3)	
Smoking status, n (%)						0.04
Never Smoker	953 (52.1)	221 (47.7)	316 (53.1)	206 (59.4)	210 (49.4)	
Former Smoker	740 (38.3)	193 (39.9)	255 (39.7)	130 (31.0)	162 (40.3)	
Current Smoker	238 (9.7)	70 (12.4)	61 (7.2)	49 (9.6)	58 (10.3)	
Comorbidity						
Hyperlipidemia, n (%)						0.23
No	342 (16.8)	107 (19.8)	111 (17.4)	58 (13.0)	66 (15.1)	
Yes	1589 (83.2)	377 (80.2)	521 (82.6)	327 (87.0)	364 (84.9)	
Hypertension, n (%)						0.001
No	417 (24.3)	119 (29.5)	137 (26.1)	89 (23.5)	72 (15.3)	
Yes	1514 (75.7)	365 (70.5)	495 (73.9)	296 (76.5)	358 (84.7)	
CVD, n (%)						0.26
No	1375 (72.9)	346 (74.1)	459 (75.6)	273 (68.7)	297 (71.0)	
Yes	556 (27.1)	138 (25.9)	173 (24.4)	112 (31.3)	133 (29.0)	
Alcohol intake, n (%)						0.24
Never	372 (18.4)	87 (17.7)	128 (20.2)	79 (17.1)	78 (17.7)	
Former	547 (25.2)	139 (26.7)	162 (20.5)	120 (30.9)	126 (26.1)	
Mild	738 (42.8)	178 (39.3)	253 (46.3)	139 (40.7)	168 (43.5)	
Moderate	159 (9.4)	53 (12.8)	55 (9.1)	20 (7.2)	31 (7.7)	
Heavy	115 (4.1)	27 (3.5)	34 (4.0)	27 (4.1)	27 (5.0)	
Physical Activity, n (%)						0.002
Inactive	983 (46.1)	217 (40.8)	313 (43.3)	196 (47.3)	257 (56.3)	
Active	948 (53.9)	267 (59.2)	319 (56.7)	189 (52.7)	173 (43.7)	
ACR category, n (%)						0.70
A1	973 (55.6)	232 (53.6)	323 (56.1)	208 (58.9)	210 (54.5)	
A2	858 (40.8)	227 (42.8)	281 (40.9)	159 (37.9)	191 (40.7)	
A3	100 (3.6)	25 (3.6)	28 (3.0)	18 (3.2)	29 (4.8)	
GFR category, n (%)						0.02
G1	158 (6.6)	41 (6.9)	40 (4.2)	36 (6.1)	41 (10.4)	
G2	488 (24.4)	126 (25.5)	170 (27.5)	89 (22.3)	103 (19.9)	
G3a	907 (50.3)	226 (51.3)	289 (49.1)	197 (54.5)	195 (47.5)	
G3b	298 (15.4)	67 (13.2)	109 (15.8)	52 (14.6)	70 (18.2)	
G4	65 (2.8)	14 (1.9)	23 (3.3)	10 (2.3)	18 (3.6)	
G5	15 (0.5)	10 (1.3)	1 (0.1)	1 (0.2)	3 (0.4)	
Mortstat, n (%)						0.01
Alive	928 (54.23)	181 (46.18)	307 (55.04)	206 (59.62)	234 (58.28)	
Deaths	1003 (45.77)	303 (53.82)	325 (44.96)	179 (40.38)	196 (41.72)	
Follow-up in years, Median (IQR)	7.6 (4.4,11.6)	7.5 (4.7,12.0)	7.8 (4.4,12.2)	8.0 (4.3,11.2)	6.7 (3.9,10.7)	0.12
Laboratory						
Albumin, (g/L), Mean ± SE	41.9 (0.1)	42.1 (0.2)	42.1 (0.1)	41.6 (0.2)	41.5 (0.2)	0.02
Hemoglobin, (g/dL), Mean ± SE	13.9 (0.0)	14.0 (0.1)	14.1 (0.1)	14.0 (0.1)	13.6 (0.1)	0.003
HEI, Mean ± SE	53.8 (0.4)	54.0 (1.0)	54.6 (0.6)	54.0 (0.8)	52.3 (1.0)	0.32
eGFR, (mL/min/1.73 m2), Mean ± SE	58.5 (0.6)	59.5 (1.0)	57.9 (0.8)	58.5 (1.2)	58.4 (1.3)	0.62
NLR, Mean ± SE	2.6 (0.0)	2.7 (0.1)	2.6 (0.1)	2.6 (0.1)	2.3 (0.1)	< 0.001

Data are represented as weighted proportion (%), mean ± SE, or median (IQR).

HbA1c, glycated hemoglobin A1c; BMI, body mass index; CVD, cardiovascular disease; eGFR, estimated glomerular filtration rate; HEI, healthy eating index; ACR, albumin-to-creatinine ratio; eGFR, estimated glomerular filtration rate; NLR, neutrophil to lymphocyte ratio.

### 3.2 HbA1c and all-cause and CVD mortality

**Figure 2:**
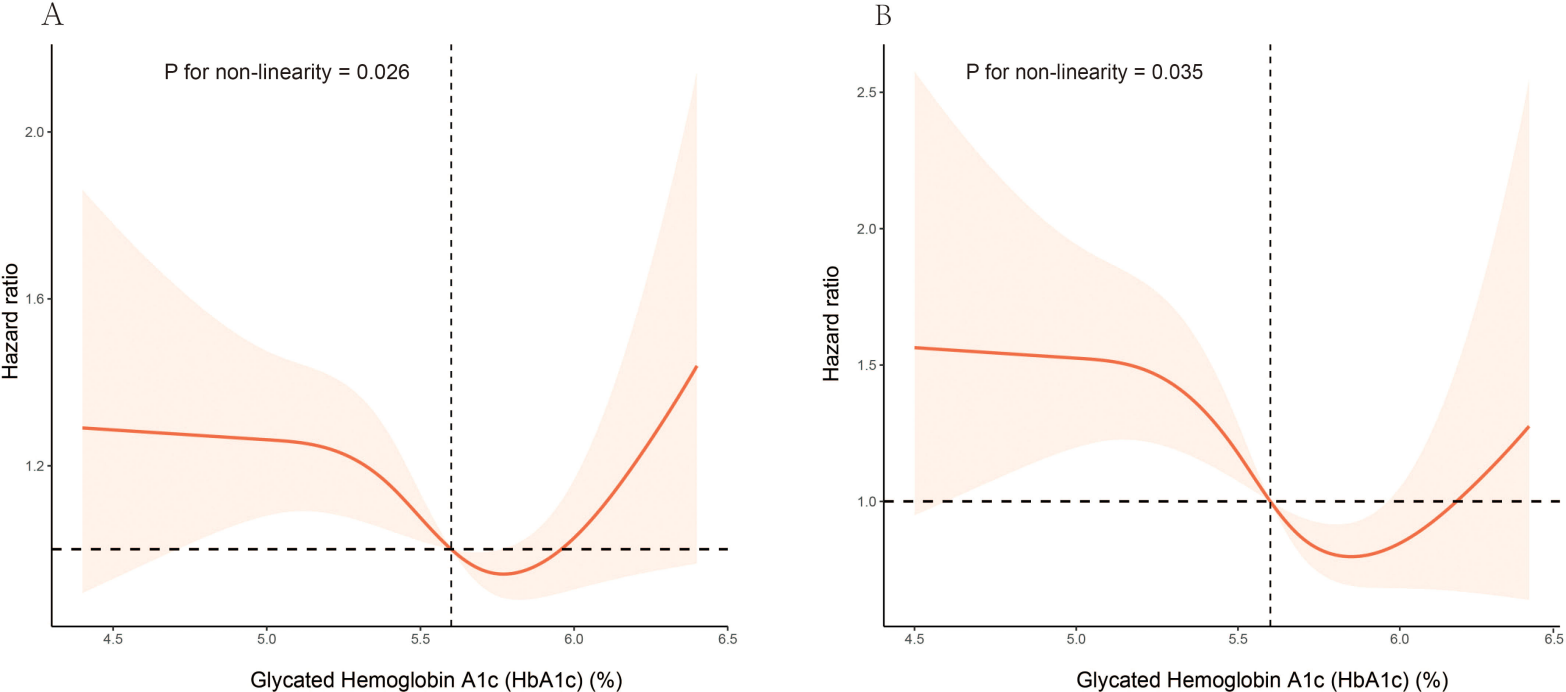
Association of HbA1c levels with all-cause and CVD mortality among elderly patients with non-diabetic chronic kidney disease in NHANES 1999 to 2018: **(A)** all-cause mortality, **(B)** CVD mortality. Hazard ratios (solid lines) and 95% CIs (shaded areas) were adjusted for age (continuous), sex (male or female), race (non-Hispanic White, non-Hispanic Black, Mexican American, or other), education (<9, 9-13, ≥13), BMI (continuous), poverty income ratio (continuous), smoking status (never, former, current), hypertension (no, yes), CVD (no, yes), hyperlipidemia (no, yes), physical activity (inactive, active), alcohol intake(never, former, mild, moderate, heavy), albumin (continuous), hemoglobin (continuous), HEI (continuous), eGFR (continuous), NLR (continuous).

In model III, the weighted multivariable Cox regression analysis revealed a non-linear association between HbA1c levels and both all-cause and CVD mortality. For all-cause mortality, compared with Quartile 3 (5.7–5.8%), the hazard ratios (HRs) for Quartile 1 (3.7–5.3%), Quartile 2 (5.4–5.6%), and Quartile 4 (5.9–6.4%) were 1.48 (95% CI: 1.18–1.87), 1.24 (95% CI: 0.98–1.57), and 1.31 (95% CI: 1.01–1.70) respectively. For CVD mortality, compared with Quartile 3, the HRs for Quartile 1, Quartile 2, and Quartile 4 were 1.94 (95% CI: 1.29–2.90), 1.49 (95% CI: 1.03–2.17), and 1.14 (95% CI: 0.73–1.77) respectively (**Table 2**). A smoothed curve fit illustrated a non-linear association between HbA1c level and both all-cause mortality (P = 0.026 for nonlinearity) and CVD mortality (P = 0.035 for nonlinearity) (**Figure 2**).

**Table 2:** Hazard ratios for all-cause and CVD mortality by Hba1c levels among elderly patients with non-diabetic chronic kidney disease in NHANES 1999 to 2018.

	Hba1c (%)				
Model	Quartile 1 (3.7-5.3)	Quartile 2 (5.4-5.6)	Quartile 3 (5.7-5.8)	Quartile 4 (5.9-6.4)	P value for trend
All-cause mortality					
Deaths/Total	303/484	325/632	179/385	196/430	
Non-adjusted Model	1.31 (1.05-1.63)	1.08 (0.88-1.33)	Ref.	1.19 (0.91-1.56)	0.66
Model I	1.42 (1.16-1.74)	1.17 (0.96-1.43)	Ref.	1.35 (1.05-1.73)	0.15
Model II	1.51 (1.18-1.94)	1.26 (1.00-1.60)	Ref.	1.33 (1.02-1.74)	0.16
Model III	1.48 (1.18-1.87)	1.24 (0.98-1.57)	Ref.	1.31 (1.01-1.70)	0.21
CVD mortality					
Deaths/Total	114/484	144/632	84/385	70/430	
Non-adjusted Model	1.25 (0.86-1.81)	1.05 (0.75-1.49)	Ref.	0.89 (0.57-1.40)	0.43
Model I	1.57 (1.11-2.23)	1.28 (0.94-1.74)	Ref.	1.11 (0.73-1.69)	0.84
Model II	1.95 (1.27-3.00)	1.57 (1.07-2.32)	Ref.	1.19 (0.75-1.89)	0.47
Model III	1.94 (1.29-2.90)	1.49 (1.03-2.17)	Ref.	1.14 (0.73-1.77)	0.67

Model I: adjusted for age (continuous), sex (male or female); Model II: adjusted for Model I plus race (non-Hispanic White, non-Hispanic Black, Mexican American, or other), education(<9, 9-13, ≥13), BMI (continuous), poverty income ratio (continuous), smoking status (never, former, current), hypertension(no, yes), CVD(no, yes), hyperlipidemia(no, yes), physical activity(inactive, active), alcohol intake(never, former, mild, moderate, heavy); Model III: adjusted for Model II plus albumin (continuous), hemoglobin (continuous), HEI (continuous), eGFR (continuous), NLR (continuous).

HbA1c, glycated hemoglobin A1c; BMI, body mass index; CVD, cardiovascular disease; eGFR, estimated glomerular filtration rate; HEI, healthy eating index; eGFR, estimated glomerular filtration rate; NLR, neutrophil to lymphocyte ratio.

### 3.3 Stratified and sensitivity analyses

Stratified analysis demonstrated consistent U-shaped associations between HbA1c levels and all-cause mortality across various strata, including sex, race/ethnicity, BMI, smoking status, hypertension, CVD, hyperlipidemia, physical activity, alcohol intake, anemia, GFR category, and ACR category. No significant interactions were observed between HbA1c levels and any stratified variable (**Table 3**). Similarly, a consistent L-shaped relationship between HbA1c levels and CVD mortality was observed across different strata, with no significant interactions detected (**Supplementary Table S1**).

**Table 3:** Associations of Hba1c levels with all-cause mortality in various subgroups among elderly patients with non-diabetic chronic kidney disease in NHANES 1999 to 2018.

Subgroup	Hba1c				P for interaction
	Quartile 1 (3.7-5.3)	Quartile 2 (5.4-5.6)	Quartile 3 (5.7-5.8)	Quartile 4 (5.9-6.4)	
	n=484	n=632	n=385	n=430	
Sex					0.15
Male	1.62 (1.12-2.35)	1.46 (1.02-2.08)	Ref.	1.66 (1.12-2.46)	
Female	1.32 (0.96-1.83)	1.06 (0.78-1.44)	Ref.	1.08 (0.76-1.55)	
Race-ethnicity					0.12
Mexican American	1.74 (0.78-3.88)	0.55 (0.30-1.03)	Ref.	1.02 (0.45-2.32)	
Non-Hispanic Black	1.11 (0.68-1.83)	0.95 (0.54-1.65)	Ref.	0.58 (0.31-1.06)	
Non-Hispanic White	1.49 (1.18-1.89)	1.27 (0.99-1.60)	Ref.	1.44 (1.07-1.93)	
Other	4.80 (1.26-18.33)	2.42 (0.75-7.84)	Ref.	1.30 (0.37-4.63)	
Poverty Income Ratio					0.23
<1.0	1.40 (0.89-2.19)	1.16 (0.77-1.76)	Ref.	1.26 (0.82-1.95)	
1.0-3.0	1.34 (1.00-1.79)	1.20 (0.85-1.68)	Ref.	1.12 (0.77-1.64)	
≥3.0	2.59 (1.54-4.35)	1.70 (1.00-2.88)	Ref.	2.48 (1.37-4.51)	
BMI- kg/m^2^					0.08
<30	1.64 (1.22-2.22)	1.34 (0.98-1.83)	Ref.	1.71 (1.25-2.33)	
≥30	1.06 (0.70-1.60)	1.17 (0.80-1.71)	Ref.	0.90 (0.58-1.39)	
Smoking Status					0.06
Never Smoker	1.68 (1.16-2.43)	1.40 (0.99-1.97)	Ref.	1.30 (0.93-1.80)	
Former Smoker	1.03 (0.74-1.42)	0.92 (0.68-1.25)	Ref.	1.18 (0.79-1.75)	
Current Smoker	3.16 (1.21-8.27)	2.08 (0.84-5.15)	Ref.	2.14 (0.85-5.40)	
Hypertension					0.2
No	0.85 (0.46-1.55)	0.85 (0.47-1.55)	Ref.	1.53 (0.84-2.76)	
Yes	1.67 (1.28-2.17)	1.33 (1.01-1.74)	Ref.	1.37 (1.03-1.83)	
Hyperlipidemia					0.51
No	0.93 (0.54-1.61)	1.03 (0.53-2.01)	Ref.	1.36 (0.68-2.71)	
Yes	1.53 (1.21-1.94)	1.26 (0.99-1.59)	Ref.	1.31 (0.99-1.72)	
CVD					0.86
No	1.53 (1.14-2.05)	1.21 (0.92-1.61)	Ref.	1.33 (0.95-1.88)	
Yes	1.25 (0.84-1.85)	1.25 (0.85-1.83)	Ref.	1.30 (0.87-1.93)	
Anemia					0.99
No	1.61 (0.90-2.87)	1.68 (0.92-3.09)	Ref.	1.60 (0.86-2.99)	
Yes	1.45 (1.12-1.87)	1.23 (0.97-1.56)	Ref.	1.31 (0.97-1.76)	
GFR category					
G1	1.45 (0.28-7.63)	0.63 (0.18-2.23)	Ref.	0.45 (0.09-2.28)	0.06
G2	1.05 (0.66-1.68)	1.01 (0.62-1.64)	Ref.	0.95 (0.52-1.75)	
G3a+G3b	1.49 (1.15-1.94)	1.31 (0.99-1.71)	Ref.	1.41 (1.05-1.89)	
G4+G5	1.93 (0.25-14.71)	0.96 (0.11-8.27)	Ref.	2.37 (0.30-18.67)	
ACR category					0.33
A1	1.48 (1.10-1.98)	1.26 (0.93-1.71)	Ref.	1.31 (0.91-1.90)	
A2	1.33 (0.90-1.96)	1.04 (0.71-1.53)	Ref.	1.07 (0.70-1.63)	
A3	0.84 (0.32-2.19)	2.07 (0.80-5.34)	Ref.	2.01 (0.82-4.95)	

Adjusted for age (continuous), sex (male or female), race (non-Hispanic White, non-Hispanic Black, Mexican American, or other), education(<9, 9-13, ≥13), BMI (continuous), poverty income ratio (continuous), smoking status (never, former, current), hypertension(no, yes), CVD(no, yes), hyperlipidemia(no, yes), physical activity(inactive, active), alcohol intake(never, former, mild, moderate, heavy), albumin (continuous), hemoglobin (continuous), HEI (continuous), eGFR (continuous), NLR (continuous). The strata variable was not included when stratifying by itself.

HbA1c, glycated hemoglobin A1c; BMI, body mass index; CVD, cardiovascular disease; eGFR, estimated glomerular filtration rate; HEI, healthy eating index; eGFR, estimated glomerular filtration rate; NLR, neutrophil to lymphocyte ratio; ACR, albumin-to-creatinine ratio.

Sensitivity analyses, excluding participants who died within 2 years of follow-up and those with an eGFR <15 mL/min/1.73 m^2^, as well as individuals who reported undergoing hemodialysis within the past year, confirmed the robustness of the results (**Supplementary Tables S2**, **S3**).

## 4 Discussion

In this comprehensive, prospective study using data from the NHANES, we investigated the correlation between HbA1c levels and mortality outcomes, both all-cause and CVD, in elderly individuals with CKD without diabetes. Our analysis revealed a nonlinear relationship between HbA1c levels and mortality risk. Our analysis revealed that lower levels of HbA1c were associated with increased mortality risk for both all-cause and cardiovascular causes, whereas elevated HbA1c levels were associated with an increased risk of all-cause mortality; however, no significant association was found with CVD mortality. Our analyses, supported by stratification and sensitivity analyses, identified an association between HbA1c levels of 5.7-5.8% and lower mortality risk in older patients with non-diabetic CKD.

HbA1c serves as a crucial marker of long–term glycemic regulation in individuals with diabetes and is significantly correlated with long–term health outcomes (21–23). Recent investigations have expanded the relevance of HbA1c levels to mortality outcomes beyond the diabetic population, suggesting their importance in non-diabetics and independence from later development of diabetes (10). An American study involving 15,792 non-diabetics underscored an L–shaped correlation between HbA1c levels and all-cause mortality (24), highlighting increased mortality associated with both low and high HbA1c levels. A similar pattern was observed in an Israeli cohort study of 12,937 non-diabetic individuals aged > 65 years (10). Our research further identifies a U–shaped correlation for older patients with non-diabetic CKD, proposing a safe HbA1c threshold of 5.7–5.8%. Contrastingly, these insights diverge from previous findings within non-diabetic CKD cohorts, although the studies by Vandana Menon et al. (13) and Claire Trivin et al. (9) demonstrated a linear relationship between HbA1c and all-cause mortality in non-diabetic CKD patients, another study on non-diabetic stage 1-4 CKD patients in Taiwan did not find this correlation (12). These disparities can be explained by various factors. First, differences in the studied populations, such as our focus on an older American cohort with a mean age of 72 years, who might possess a higher burden of chronic conditions, thereby elevating mortality risk, might have influenced the outcomes. Moreover, racial variations in HbA1c levels (25) and their impact on CKD progression (26) emerge as potential confounders. Although our study’s race–stratified analysis yielded uniform results across subgroups, it did not wholly account for the observed discrepancies. Additionally, variations in the study methodologies and data acquisition approaches could account for these disparate findings. Our investigation employed a prospective cohort design, leveraging the U.S. national population via the NHANES, in contrast to other studies that might rely on hospital–based data (9, 12) or retrospective analyses of distinct cohorts (9, 13). Finally, the use of diverse statistical approaches and analytical models, including our utilization of a multivariable adjustment framework and restricted cubic spline analysis to clarify the nonlinear dynamics between HbA1c and mortality, may also have played a role in the divergent results.

This investigation reaffirmed the link between elevated HbA1c levels and increased all-cause mortality among non-diabetic individuals, a finding that is in line with that of prior research. Elevated HbA1c has been implicated in the advancement of CKD and has been acknowledged as an independent predictor of augmented arterial stiffness in elderly patients with non-diabetic kidney disease (27). Beyond its link to CKD, the association observed between elevated HbA1c levels and increased all-cause mortality may be partially explained through cancer-related pathways. Previous studies have demonstrated that disrupted glucose metabolism is associated with increased cancer risk and cancer-related mortality (28–30). This biological link between dysglycemia and cancer outcomes provides a potential mechanism underlying our observed association between elevated HbA1c and increased all-cause mortality. Our study did not reveal a significant association between high HbA1c levels and mortality from CVD, potentially due to the limited statistical power resulting from the relatively small number of CVD cases. This limitation highlights the need for future studies that are more comprehensive or longer in duration to fully assess the effect of HbA1c levels on CVD progression.

Remarkably, our analysis revealed that lower HbA1c levels are associated with increased mortality from all causes and cardiovascular issues in non-diabetic elderly patients with CKD —a novel finding within this demographic, previously documented in diabetic populations (23). In a substantial study involving 243,222 U.S. veterans, hypoglycemia was found to occur frequently among patients with CKD, irrespective of their diabetic status (31). This phenomenon can likely be attributed to diminished renal function and changes in insulin metabolism within the kidneys, such as reduced clearance of endogenous insulin by both the kidneys and liver. Furthermore, low HbA1c levels might indicate malnutrition, a condition particularly common in individuals with CKD and often associated with protein–energy wasting, a critical factor that can accelerate the progression of CKD (32). In addition, lower HbA1c levels are linked to frailty in elderly individuals (33), a condition that often leads to death, with weight loss playing a central role in this association. In our study, we found a correlation between lower HbA1c levels and reduced BMI. However, our stratified analyses across different BMI categories revealed consistent outcomes, indicating that low body weight does not solely account for increased mortality risk. This suggests that multiple factors may explain the relationship between low HbA1c levels and higher mortality risk. It’s worth mentioning that concerns have been raised about the potential impact of chronic renal failure on the accuracy of HbA1c measurements due to factors such as shortened lifespan of erythrocytes and increased levels of carbamylated hemoglobin, which could falsely influence this association (34–36). To address these concerns, we conducted a stratified analysis that considered anemia and renal disease stages. The results remained unchanged and consistent even after excluding patients with end–stage CKD and those undergoing hemodialysis, which could affect the HbA1c values in these individuals. Therefore, we conclude that these factors did not significantly compromise the reliability of our findings. Evidence indicates that carbamylated hemoglobin constitutes only a minor portion of HbA1c in patients on dialysis (37), supporting the stability of these findings.

This comprehensive population–based investigation has several notable strengths. Primarily, it utilizes a nationally representative sample, which facilitates the extrapolation of the findings to a broader United States population. The extensive follow–up duration of 7.6 years endows the study with a solid foundation to assess the enduring association between HbA1c levels and mortality. This extended period allowed for a thorough examination of the stability of this relationship over time. Furthermore, the meticulous matching and low discrepancy rate in the NHANES-linked mortality data augment the credibility of the study. Using the extensive dataset provided by NHANES, this study adeptly managed potential confounders encompassing demographic, socioeconomic, lifestyle, and dietary factors, thus enhancing the study’s internal validity. The sizable sample provides the statistical power necessary for conducting nuanced joint and stratified analyses, delving deeper into the relationship between HbA1c and all-cause mortality.

Nonetheless, this study had certain limitations. The absence of longitudinal data on the diabetes status precludes a comprehensive assessment of the potential impact of diabetes progression on the conclusions of this study. Furthermore, due to the cross-sectional nature of NHANES measurements, we were unable to distinguish between acute and chronic changes in eGFR and ACR. This single-time-point assessment might not fully represent the typical CKD population. Moreover, CKD–associated variations can potentially influence erythrocyte turnover or glycation processes, thus affecting HbA1c measurements. Despite the stability of the outcomes of the sensitivity analyses, this potential interference could not be ruled out entirely. Additionally, due to the NHANES database limitations, we were unable to obtain information about the primary causes of kidney disease in our study population. This heterogeneity in underlying kidney disease etiology could potentially influence the relationship between HbA1c and mortality outcomes. Furthermore, given that the analysis targeted non-diabetic individuals over the age of 60 years, the applicability of the findings may be confined to this specific group. This necessitates a cautious approach when extending these results to different populations, considering their unique characteristics and potential variances.

In summary, this observational study demonstrates a complex association between HbA1c levels and mortality risk in non-diabetic elderly individuals with CKD. Our findings reveal a U-shaped association between HbA1c levels and all-cause mortality, with both elevated and low HbA1c levels being associated with increased mortality risk in this population. These associations, while noteworthy, should be interpreted within the context of an observational study design. Further interventional studies are needed to understand the clinical implications of these findings.

## Data Availability

The datasets presented in this study can be found in online repositories. The names of the repository/repositories and accession number(s) can be found below: https://wwwn.cdc.gov/nchs/nhanes.
