# Supplementary material for: Nonlinear association between glycated hemoglobin levels and mortality in elderly patients with non-diabetic chronic kidney disease: a national health and nutrition examination survey analysis
Source: Front Endocrinol (Lausanne). 2025 Feb 11;16:1416506. doi: 10.3389/fendo.2025.1416506 (PMC11850242; doi:10.3389/fendo.2025.1416506)
Supplement: Supplementary file 1 [file Table1.docx]

Supplementary Material

Nonlinear Association Between Glycated Hemoglobin Levels and Mortality in Elderly Patients with Non-Diabetic Chronic Kidney Disease: A National Health and Nutrition Examination Survey Analysis

Lihua Huang^1†^, Liuliu He^1†^, Qingfeng Zeng^2^, Jinjing Huang^3^, Xiaoyan Luo^4^, Qiuming Zhong^5*^

***Correspondence: Qiuming Zhong: 609277033@qq.com**

# Supplementary Figures and Tables

**eTable 1.** Associations of Hba1c levels with CVD mortality in various subgroups among elderly patients with non-diabetic chronic kidney disease in NHANES 1999 to 2018

| **Subgroup** | **Hba1c** | | | | **P for interaction** |
| --- | --- | --- | --- | --- | --- |
|  | **Quartile 1 (3.7-5.3)** | **Quartile 2 (5.4-5.6)** | **Quartile 3 (5.7-5.8)** | **Quartile 4 (5.9-6.4)** |  |
|  | **n=484** | **n=632** | **n=385** | **n=430** |  |
| Sex |  |  |  |  | 0.53 |
| Male | 1.69 (1.04-2.75) | 1.18 (0.73-1.93) | Ref. | 1.30 (0.68-2.49) |  |
| Female | 1.95 (1.07-3.54) | 1.41 (0.81-2.47) | Ref. | 0.94 (0.52-1.71) |  |
| Race-ethnicity |  |  |  |  | 0..11 |
| Mexican American | 1.72 (0.37-7.93) | 0.30 (0.04-2.40) | Ref. | 1.46 (0.37-5.70) |  |
| Non-Hispanic Black | 0.83 (0.31-2.24) | 1.14 (0.50-2.59) | Ref. | 0.38 (0.11-1.26) |  |
| Non-Hispanic White | 2.08 (1.35-3.21) | 1.40 (0.93-2.11) | Ref. | 1.22 (0.75-1.98) |  |
| Other | 0.66 (0.07-6.50) | 1.13 (0.34-3.69) | Ref. | 0.35 (0.05-2.26) |  |
| Poverty Income Ratio |  |  |  |  | 0.15 |
| <1.0 | 1.53 (0.72-3.26) | 1.19 (0.71-1.99) | Ref. | 0.83 (0.40-1.72) |  |
| 1.0-3.0 | 1.55 (0.98-2.46) | 1.15 (0.62-2.14) | Ref. | 0.79 (0.42-1.47) |  |
| ≥3.0 | 4.25 (1.94-9.30) | 2.65 (1.06-6.61) | Ref. | 3.75 (1.45-9.69) |  |
| BMI- kg/m^2^ |  |  |  |  | 0.14 |
| <30 | 1.78 (1.1-2.89) | 1.38 (0.9-2.14) | Ref. | 1.52 (0.92-2.5) |  |
| ≥30 | 1.50 (0.77-2.94) | 1.28 (0.67-2.42) | Ref. | 0.63 (0.29-1.34) |  |
| Smoking Status |  |  |  |  | 0.07 |
| Never Smoker | 2.01 (1.08-3.74) | 1.67 (0.95-2.95) | Ref. | 0.91 (0.52-1.59) |  |
| Former Smoker | 1.34 (0.80-2.25) | 0.75 (0.44-1.31) | Ref. | 1.27 (0.69-2.34) |  |
| Current Smoker | 6.05 (1.62-22.63) | 2.28 (0.6-8.63) | Ref. | 2.19 (0.41-11.63) |  |
| Hypertension |  |  |  |  | 0.09 |
| No | 0.75 (0.28-2.00) | 0.62 (0.21-1.81) | Ref. | 1.65 (0.58-4.68) |  |
| Yes | 2.13 (1.35-3.34) | 1.49 (0.96-2.32) | Ref. | 1.05 (0.67-1.66) |  |
| Hyperlipidemia |  |  |  |  | 0.74 |
| No | 1.08 (0.38-3.07) | 1.22 (0.52-2.85) | Ref. | 2.25 (0.78-6.53) |  |
| Yes | 2.00 (1.35-2.96) | 1.45 (1.02-2.06) | Ref. | 1.13 (0.75-1.72) |  |
| CVD |  |  |  |  | 0.48 |
| No | 1.53 (0.93-2.52) | 1.11 (0.70-1.78) | Ref. | 0.92 (0.49-1.7) |  |
| Yes | 2.10 (1.17-3.77) | 1.93 (1.18-3.15) | Ref. | 1.49 (0.80-2.78) |  |
| Anemia |  |  |  |  | 0.26 |
| No | 1.85 (1.15-2.98) | 1.44 (0.95-2.16) | Ref. | 0.99 (0.59-1.67) |  |
| Yes | 1.46 (0.58-3.71) | 1.64 (0.67-4.00) | Ref. | 3.03 (1.14-8.07) |  |
| GFR category |  |  |  |  |  |
| G1 | 33.93 (12.73-90.45) | 0.25 (0.03-2.05) | Ref. | 0.02 (0.01-0.03) | 0.62 |
| G2 | 1.12 (0.45-2.77) | 0.89 (0.36-2.22) | Ref. | 0.82 (0.26-2.57) |  |
| G3a+G3b | 2.01 (1.32-3.07) | 1.36 (0.94-1.98) | Ref. | 1.03 (0.65-1.62) |  |
| G4+G5 | NE | NE | Ref. | NE |  |
| ACR category |  |  |  |  | 0.27 |
| A1 | 2.29 (1.43-3.68) | 1.45 (0.92-2.29) | Ref. | 1.27 (0.72-2.23) |  |
| A2 | 1.46 (0.74-2.88) | 1.08 (0.58-2.01) | Ref. | 0.70 (0.32-1.57) |  |
| A3 | NE | NE | Ref. | NE |  |

**Notes:** adjusted for age (continuous), sex (male or female), race (non-Hispanic White, non-Hispanic Black, Mexican American, or other), education(<9, 9-13, ≥13), BMI (continuous), poverty income ratio (continuous), smoking status (never, former, current), hypertension(no, yes), CVD(no, yes), hyperlipidemia(no, yes), physical activity(inactive, active), alcohol intake(never, former, mild, moderate, heavy), albumin (continuous), hemoglobin (continuous), HEI (continuous), eGFR (continuous), NLR (continuous). The strata variable was not included when stratifying by itself.

**Abbreviations:** NE, not evaluable; HbA1c, glycated hemoglobin A1c; BMI, body mass index; CVD, cardiovascular disease; eGFR, estimated glomerular filtration rate; HEI, healthy eating index; ACR, albumin-to-creatinine ratio; eGFR, estimated glomerular filtration rate; NLR, neutrophil to lymphocyte ratio; ACR, albumin-to-creatinine ratio

**eTable 2.** Hazard Ratios for all-Cause and CVD mortality among elderly patients with non-diabetic chronic kidney disease after excluding participants who died within two years of follow-up in NHANES 1999–2018 (n=156)

|  | **Hba1c (%)** | | | |  |
| --- | --- | --- | --- | --- | --- |
| **Model** | **Quartile 1 (3.7-5.3)** | **Quartile 2 (5.4-5.6)** | **Quartile 3 (5.7-5.8)** | **Quartile 4 (5.9-6.4)** | **P value for trend** |
| **All-cause mortality** |  |  |  |  |  |
| Deaths/Total | 273/444 | 295/585 | 161/350 | 170/396 |  |
| Non-adjusted Model | 1.31 (1.04-1.66) | 1.08 (0.87-1.34) | Ref. | 1.16 (0.86-1.54) | 0.86 |
| Model Ⅰ | 1.44 (1.16-1.80) | 1.18 (0.96-1.46) | Ref. | 1.33 (1.01-1.74) | 0.23 |
| Model Ⅱ | 1.55 (1.19-2.03) | 1.28 (0.99-1.66) | Ref. | 1.32 (0.99-1.76) | 0.24 |
| Model Ⅲ | 1.52 (1.19-1.96) | 1.26 (0.97-1.63) | Ref. | 1.29 (0.98-1.70) | 0.30 |
| **CVD mortality** |  |  |  |  |  |
| Deaths/Total | 102/444 | 130/585 | 73/350 | 59/396 |  |
| Non-adjusted Model | 1.32 (0.89-1.95) | 1.13 (0.80-1.60) | Ref. | 0.89 (0.54-1.46) | 0.48 |
| Model Ⅰ | 1.70 (1.17-2.46) | 1.40 (1.02-1.92) | Ref. | 1.12 (0.70-1.79) | 0.74 |
| Model Ⅱ | 2.17 (1.39-3.40) | 1.74 (1.16-2.61) | Ref. | 1.20 (0.72-1.99) | 0.44 |
| Model Ⅲ | 2.15 (1.40-3.29) | 1.64 (1.11-2.41) | Ref. | 1.15 (0.71-1.86) | 0.64 |

**Notes:** Model I: adjusted for age (continuous), sex (male or female); Model Ⅱ: adjusted for Model Ⅰ plus race (non-Hispanic White, non-Hispanic Black, Mexican American, or other) , education(<9, 9-13, ≥13), BMI (continuous), poverty income ratio (continuous), smoking status (never, former, current), hypertension(no, yes), CVD(no, yes), hyperlipidemia(no, yes), physical activity(inactive, active), alcohol intake(never, former, mild, moderate, heavy); Model Ⅲ: adjusted for Model Ⅱ plus albumin (continuous), hemoglobin (continuous), HEI (continuous), eGFR (continuous), NLR (continuous).

**Abbreviations:** HbA1c, glycated hemoglobin A1c; BMI, body mass index; CVD, cardiovascular disease; eGFR, estimated glomerular filtration rate; HEI, healthy eating index; eGFR, estimated glomerular filtration rate; NLR, neutrophil to lymphocyte ratio

**eTable 3.** Hazard Ratios for all-cause and CVD Mortality among elderly patients with non-diabetic chronic kidney disease after excluding participants who self-reported receiving hemodialysis within the past year or had an eGFR < 15 mL/min/1.73 m² in NHANES 1999–2018 (n=18)

|  | **Hba1c (%)** | | | |  |
| --- | --- | --- | --- | --- | --- |
| **Model** | **Quartile 1 (3.7-5.3)** | **Quartile 2 (5.4-5.6)** | **Quartile 3 (5.7-5.8)** | **Quartile 4 (5.9-6.4)** | **P value for trend** |
| **All-cause mortality** |  |  |  |  |  |
| Deaths/Total | 295/473 | 324/630 | 179/384 | 193/426 |  |
| Non-adjusted Model | 1.29 (1.03-1.61) | 1.08 (0.88-1.33) | Ref. | 1.18 (0.90-1.55) | 0.65 |
| Model Ⅰ | 1.40 (1.14-1.71) | 1.17 (0.96-1.43) | Ref. | 1.34 (1.04-1.72) | 0.14 |
| Model Ⅱ | 1.48 (1.16-1.90) | 1.26 (1.00-1.59) | Ref. | 1.33 (1.02-1.73) | 0.15 |
| Model Ⅲ | 1.45 (1.16-1.83) | 1.24 (0.98-1.56) | Ref. | 1.30 (1.01-1.69) | 0.19 |
| **CVD mortality** |  |  |  |  |  |
| Deaths/Total | 111/473 | 144/630 | 84/384 | 69/426 |  |
| Non-adjusted Model | 1.22 (0.84-1.78) | 1.05 (0.75-1.49) | Ref. | 0.88 (0.56-1.39) | 0.44 |
| Model Ⅰ | 1.54 (1.09-2.19) | 1.28 (0.94-1.74) | Ref. | 1.10 (0.72-1.68) | 0.83 |
| Model Ⅱ | 1.92 (1.25-2.94) | 1.57 (1.07-2.32) | Ref. | 1.17 (0.74-1.86) | 0.48 |
| Model Ⅲ | 1.89 (1.27-2.82) | 1.49 (1.03-2.16) | Ref. | 1.12 (0.72-1.73) | 0.69 |

**Notes:** Model I: adjusted for age (continuous), sex (male or female); Model Ⅱ: adjusted for Model Ⅰ plus race (non-Hispanic White, non-Hispanic Black, Mexican American, or other) , education(<9, 9-13, ≥13), BMI (continuous), poverty income ratio (continuous), smoking status (never, former, current), hypertension(no, yes), CVD(no, yes), hyperlipidemia(no, yes), physical activity(inactive, active), alcohol intake(never, former, mild, moderate, heavy); Model Ⅲ: adjusted for Model Ⅱ plus albumin (continuous), hemoglobin (continuous), HEI (continuous), eGFR (continuous), NLR (continuous).

**Abbreviations:** HbA1c, glycated hemoglobin A1c; BMI, body mass index; CVD, cardiovascular disease; eGFR, estimated glomerular filtration rate; HEI, healthy eating index; eGFR, estimated glomerular filtration rate; NLR, neutrophil to lymphocyte ratio
